# Reduced serum levels of pro-inflammatory chemokines in fragile X syndrome

**DOI:** 10.1186/s12883-020-01715-2

**Published:** 2020-04-15

**Authors:** Anke Van Dijck, Susana Barbosa, Patricia Bermudez-Martin, Olfa Khalfallah, Cyprien Gilet, Emanuela Martinuzzi, Ellen Elinck, R. Frank Kooy, Nicolas Glaichenhaus, Laetitia Davidovic

**Affiliations:** 1 Department of Medical Genetics, University and University Hospital of Antwerp, Edegem, Belgium; 2 Université Côte d’Azur, CNRS, Institut de Pharmacologie Moléculaire et Cellulaire, Valbonne, France; 3 Université Côte d’Azur, CNRS, Laboratoire Informatique Signaux et Systèmes de Sophia Antipolis, Valbonne, France

**Keywords:** Fragile X syndrome, Cytokines, Chemokines, Biomarkers, Clustering, Autism spectrum disorders, Intellectual disability

## Abstract

**Background:**

Fragile X syndrome (FXS) is the most frequent cause of inherited intellectual disability and the most commonly identified monogenic cause of autism. Recent studies have shown that long-term pathological consequences of FXS are not solely confined to the central nervous system (CNS) but rather extend to other physiological dysfunctions in peripheral organs. To gain insights into possible immune dysfunctions in FXS, we profiled a large panel of immune-related biomarkers in the serum of FXS patients and healthy controls.

**Methods:**

We have used a sensitive and robust Electro Chemi Luminescence (ECL)-based immunoassay to measure the levels of 52 cytokines in the serum of *n* = 25 FXS patients and *n* = 29 healthy controls. We then used univariate statistics and multivariate analysis, as well as an advanced unsupervised clustering method, to identify combinations of immune-related biomarkers that could discriminate FXS patients from healthy individuals.

**Results:**

While the majority of the tested cytokines were present at similar levels in FXS patients and healthy individuals, nine chemokines, CCL2, CCL3, CCL4, CCL11, CCL13, CCL17, CCL22, CCL26 and CXCL10, were present at much lower levels in FXS patients. Using robust regression, we show that six of these biomarkers (CCL2, CCL3, CCL11, CCL22, CCL26 and CXCL10) were negatively associated with FXS diagnosis. Finally, applying the K-sparse unsupervised clustering method to the biomarker dataset allowed for the identification of two subsets of individuals, which essentially matched the FXS and healthy control categories.

**Conclusions:**

Our data show that FXS patients exhibit reduced serum levels of several chemokines and may therefore exhibit impaired immune responses. The present study also highlights the power of unsupervised clustering methods to identify combinations of biomarkers for diagnosis and prognosis in medicine.

## Background

Fragile X syndrome (FXS) is the most frequent cause of inherited intellectual disability (ID) and the most commonly identified monogenic cause of autism spectrum disorders (ASD) [1, 2]. This X-linked genetic disease is caused by the silencing of the Fragile X Mental Retardation 1 (*FMR1*) gene positioned in Xq27.3 [3]. FXS is more prevalent in males (1.4:1000) than in females (0.9/10,000) and females tend to display milder impairments [4]. In FXS patients, a dynamic mutation abnormally increases the number of CGG repeats in the first exon of the *FMR1* gene, above the premutation threshold of 200 repeats, leading to their hypermethylation and the subsequent absence of its gene product FMRP, an RNA-binding protein controlling translation [5, 6]. Although a monogenic disorder, FXS is a disease of complex aetiology. FXS prominent phenotypes notably include intellectual disability, epilepsy, anxiety linked to sensory hyperarousal, attention deficits and hyperactivity disorder [7]. Furthermore, stereotypies and social interaction deficits lead to ASD diagnosis in 60% of boys and 25% of girls with FXS [8]. FXS is also accompanied by physical and anatomical abnormalities (macroorchidism, elongated face, hyperextensible finger joints) and recurrent health problems such as gastro-intestinal dysfunctions [7, 9]. Recurrent media otites are frequent in FXS patients (52.6% versus 12.6% in neurotypical controls), as well sinusitis, although the incidence of other types of infections is not different in FXS patients [9, 10]. This suggests that the long-term pathological consequences of this neurodevelopmental disorder are not solely confined to the central nervous system and could extend to other physiological dysfunctions in peripheral systems.

To study possible peripheral consequences of *FMR1-*deficiency, some authors have compared the levels of serum and plasma biomarkers between FXS patients and healthy controls. Prior studies have focused on metabolic markers and have highlighted reduced levels of cholesterol [11–13] and abnormal abundances of the metabolic hormones leptin and adiponectin [14]. In a translational study addressing metabolic consequences of *FMR1*-deficiency both in a FXS mouse model and in FXS patients, we have recently shown that FXS patients display reductions in circulating glucose and increases in both free fatty acids and insulin, underlining metabolic anomalies in FXS [15]. Regarding possible immune dysfunctions in FXS, only a few studies have characterized FXS patients for immune biomarkers such as cytokines. These small circulating molecules are secreted by both immune and non-immune cell types and include notably interleukins (IL), chemokines, interferons (IFN) and members of the Tumour Necrosis Factor (TNF) family. Cytokines regulate the differentiation, activation and effector function of all immune cell types [16]. While inflammatory chemokines attract immune cells to inflammatory sites, homeostatic chemokines control cell migration during development. To our knowledge, only two studies have investigated possible immune dysfunction in FXS patients. In a first study, Ashwood et al. have assessed plasma samples from FXS male patients (*n* = 40 FXS with ASD; *n* = 64 FXS without ASD) and typically developping controls (*n* = 19) for the levels of 22 cytokines. Compared to healthy controls, the authors found that FXS patients exhibited higher plasma levels of the pro-inflammatory cytokine IL-1α and IL-12p40, and lower levels of the chemokines CCL2, CCL5, CCL11 and CXCL10 [17]. In a second study, Careaga et al. compared peripheral blood mononuclear cells (PBMC) from FXS children (*n* = 27) and healthy age-matched individuals (*n* = 8) for their ability to secrete pro-inflammatory cytokines in response to lipopolysaccharides (LPS) and Phytohemagglutin (PHA) [18]. While the basal immune responsiveness of PBMC to LPS and PHA was not impacted, PBMC from FXS patients secreted higher levels of the pro-inflammatory cytokines IL-6 and IL-12p40 compared to those from healthy controls in the presence of the group I mGluR agonist DHPG. Taken together, these two studies suggested that FXS patients may exhibit immune dysfunction. To further investigate this issue, we have used a highly sensitive and robust multiplex immunoassay to assess serum samples from FXS patients (*n* = 25) and age- and sex-matched controls (*n* = 29) for the level of 52 immune-related biomarkers. We then analysed this dataset using both standard univariate statistical method, robust Elastic Net regression and an advanced unsupervised clustering method.

## Methods

### Study sample

This study entitled “Identification of Fragile X Syndrome soluble biomarkers” was approved by the medical ethics committee of the University of Antwerp in Belgium Protocol agreement #: B300201523589) and conducted in accordance with statutes and regulations regarding the protection of the right and welfare of human subjects’ participation in biomedical research (World Declaration of Helsinki). In a previous study which found significant alterations in circulating cytokine levels in FXS patients compared to healthy controls [17], the exact effect sizes were not indicated and we therefore could not use previous knowledge to compute power. We therefore conducted a power analysis to determine the sample size required to identify differences for a theoretical large effect size (Cohen’s δ > 0.75) with a power of 80% and an error rate set at 5%. Since we had no a priori knowledge regarding the normality of the to be obtained data, we computed the sample sizes under two comparison conditions and found that *n* = 26 individuals/group would be required for Student’s T-test comparisons, while *n* = 30/group would be required for Mann & Whitney non-parametric comparisons between the two groups. Based on this, we aimed to recruit *n* = 30 patients and *n* = 30 matching controls, at the Centre for Medical Genetics of the University of Antwerp (Antwerp, Belgium), at which patients are regularly received for a yearly consultation.

As a result, 29 healthy subjects (24 males, 5 females) and 25 fragile X patients (20 males, 5 females) of matching ages and ethnicity were enrolled (Table 1). The absence/presence of the fragile X mutation was confirmed in all participants by an accredited laboratory, using a CGG-repeat PCR and Southern Blotting on DNA isolated from blood. Inclusion criteria were age 6–18 and a stable medication regimen for the previous 8 weeks. Out of the 54 subjects enrolled, 2 FXS patients were actually under Ritaline treatment (X12 and X25) and were advised not to take the drug on the day of sampling. The other subjects were not under any type of medication. Clinical examination and parental questionnaire confirmed that none of the enrolled subjects presented with a recent infection episode on the day of sampling. Exclusion criteria were: recent history of seizure, epilepsy, blackouts, clinically unstable medical disease, progressive CNS disease/disorder, history of psychiatric disorders, behavioural dysfunction to the point that subject cannot cooperate for testing and history of pathologies which could modify blood biochemistry. Written informed consent was obtained from each participant or his/her legal guardian before research participation. To avoid stress induced by fasting to Fragile X patients, individuals involved in the study were not advised to fast prior sampling. Blood was withdrawn in serum collection tubes (BD Vacutainer Serum Separator Tubes), incubated for at least 30 min at room temperature then centrifuged at 2000 rpm, 10 min, at room temperature. Serum was collected, aliquoted and immediately snapped-frozen in liquid nitrogen prior storage at − 80 °C until use. Information regarding sex, age, body mass index (BMI) and time of blood sampling were also retrieved.

**Table 1:** Characteristics of FXS patients and healthy controls

ID	Sex	Age ^#^	BMI (kg/m^2^)	Time of sampling
**Controls**				
C1	Male		17.4	15:10
C2	Female		16.9	13:40
C3	Male		16.9	13:44
C4	Male		17.9	16:57
C5	Male		14.4	11:10
C6	Male		20.1	07:40
C7	Male		19.4	10:28
C8	Female		18.9	14:05
C9	Female		19.9	13:55
C10	Male		18.0	10:40
C11	Male		21.4	16:20
C12	Male		23.5	14:45
C13	Male	Age range	22.1	13:40
C14	Female	of control	15.4	08:00
C15	Male	group:	21.4	07:15
C16	Male	5.7–19.3 yrs	25.2	07:15
C17	Male		22.4	07:40
C18	Male		14.6	17:00
C19	Male		16.4	17:38
C20	Male		20.2	17:20
C21	Male		22.2	17:26
C22	Male		15.4	16:45
C23	Male		16.4	17:05
C24	Male		14.8	16:00
C25	Male		13.9	16:10
C26	Female		18.9	15:40
C27	Male		13.8	12:53
C28	Male		17.1	06:45
C29	Male		19.6	07:45
**FXS Patients**				
X1	Male		16.3	15:45
X2	Male		18.1	12:10
X3	Male		16.3	16:20
X4	Male		23.7	11:00
X5	Male		16.9	15:20
X6	Male		21.2	12:00
X7	Male		16.4	09:00
X8	Male		16.7	14:00
X9	Male		15.9	11:45
X10	Male		13.0	19:30
X11	Male	Age range	14.1	11:30
X12*	Male	of FXS	15.0	11:50
X13	Female	group:	18.4	10:45
X14	Male	6.3–17.9 yrs	16.9	10:55
X15	Male		18.3	10:15
X16	Female		19.5	10:20
X17	Female		34.5	07:40
X18	Male		15.9	13:25
X19	Male		15.8	10:45
X20	Male		38.8	19:30
X21	Male		19.0	09:20
X22	Male		20.1	13:50
X23	Female		24.8	09:15
X24	Male		18.9	09:10
X25*	Female		20.3	10:30
Control	82.7%	12.0	18.0	13:55
FXS	80.0%	12.5	18.1	11:30
*p*-value	1	0.3769	0.8803	0.2527

For the sex variable, percentage of males in each group and p-value for Fisher’s exact test are indicated. For the other variables, range or medians of each group and *p*-values for Mann & Whitney tests are indicated. Patients under treatment are labelled with *. (#) To preserve anonymity, instead of individual ages, age ranges are specified for each group

### Biomarkers measurements

Serum samples were assessed for biomarker levels using the V-plex® kits for the Meso Scale Discovery (MSD) analytical platform, according to manufacturer’s instructions. We used the following kits: Human Cytokine 30-Plex, V-PLEX Plus Th17 Panel 1, Human Chemokine Panel 2 and Vascular Injury Panel 2 to measure immune-related biomarkers: CCL1, CCL2, CCL3, CCL4, CCL11, CCL13, CCL15, CCL17, CCL19, CCL20, CCL22, CCL26, CCL27, CXCL1, CXCL5, CXCL10, CXCL11, CXCL12, CX3CL1, GM-CSF, IFN-γ, IL-1α, IL-1β, IL-2, IL-4, IL-5, IL-6, IL-7, IL-8, IL-10, IL-12p40, IL-12p70, IL-13, IL-15, IL-16, IL-17A, IL-17B, IL-17E, IL-17A/F, IL-21, IL-22, IL-23, IL-27, IL-31, IL-33, TNF-α, TNF-β, C-reactive protein (CRP), Macrophage Migration Inhibitory Factor (MIF), SAA, sICAM-1, sVCAM-1 and VEGF-A. All 54 samples (sera from *n* = 29 controls and *n* = 25 FXS patients) were systematically analysed on the same plate to limit measurements biases. The concentration of each marker was calculated using the MSD software. The 0 value was imputed for concentrations which appeared below the lower limit of detection as determined by the MSD software.

### Statistical analysis

The distribution of biomarkers levels appeared non-normal in both groups, even when log-transformation was applied. Therefore, comparison analyses were performed using the non-parametric Mann & Whitney U-test with Bonferroni’s multiple test correction. Statistical significance was set according to a corrected *p-*value (*p*) < 0.05. Only significant differences are displayed on the graphs. We computed the r effect size statistic for the Mann–Whitney U-test, which corresponds to the *Z* value from the test divided by the total number of observations. Statistics of effect size for the Mann–Whitney U-test assesses the degree to which one group has data with higher ranks compared to the other group and unlike *p*-values, they are not affected by sample size.

Elastic Net logistic regression models were implemented with the aim of performing variable selection, leading to sparser final model, in agreement with previous recommendations [19]. To study the association between 9 dysregulated chemokines identified by univariate analysis (CCL2, CCL3, CCL4, CCL11, CCL13, CCL17, CCL22, CCL26 and CXCL10) and FXS diagnosis, adjusting for age, BMI and time of sampling, we used robust regression with 2000 subsampling steps. We used the recent R package *enetLTS*: Robust and Sparse Methods for High Dimensional Linear and Logistic Regression developed by Kurnaz et al. [20, 21]. As this method currently does not handle categorical variables such as sex, the analysis was also rerun on a reduced dataset consisting of male subjects only.

Correlations between cytokine pairs identified in the clustering analysis were computed using the Spearman’s ρ correlation coefficient rank test with Benjamini & Hochberg’s multiple test correction.

Statistical analyses were performed using R and graphs generated using GraphPad Prism version 6.00 for iOS (GraphPad Software, USA).

### Over-representation analysis

To generate the chemokine receptor interaction table (Additional file 1: Fig. S1), we used the InnateDB database [22], the expert-curated reference database in the IUPHAR/BPS Guide to Pharmacology [23] and a recent review of literature [16]. The webtool from the InnateDB database [22] was used to determine over-represented pathways within the set of 9 dysregulated chemokines.

### Clustering analysis

Clustering analysis was performed using K-sparse algorithm [24]. K-sparse clustering aims at identifying discriminated clusters, by transforming the space of the features’ (biomarkers concentration) and removing the features which appear the less pertinent for the identification of clusters. As an input dataset, we enter the raw values of concentrations for each feature and each subject in the K-sparse algorithm. A pre-processing step is embedded in the K-sparse algorithm, which allows the automated normalization of the features to guarantee the convergence of the algorithm, as described in [24]. Then, for some initial labels *Y*, the first step of K-sparse computes a weighted projection matrix *W* which linearly combines the features such that most samples are projected close to the centroid of their associated cluster. The clustering computed by K-sparse therefore considers the possible presence of outliers or extreme values through the weighted linear combination of the selected features. A sparsity constraint on *W* allows removal of features which are not sufficiently relevant to discriminate these clusters according to the fixed labels *Y*. The second step aims to fix this optimized weighted matrix *W* and to minimize the Within Cluster Sum of Squares (WCSS) by running the *k*-means algorithm in the projected space. In other words, this second step aims to update the labels according to the previously weighted and combined features. By repeating these two alternating steps, K-sparse algorithm converges to a solution. Finally, the t-Distributed Stochastic Neighbour Embedding (*t-SNE*) method is used to render a graphical output of clustering. Complete code of the K-sparse algorithm will be made available upon request.

## Results

### Immune markers analysis

**Fig. 1:**
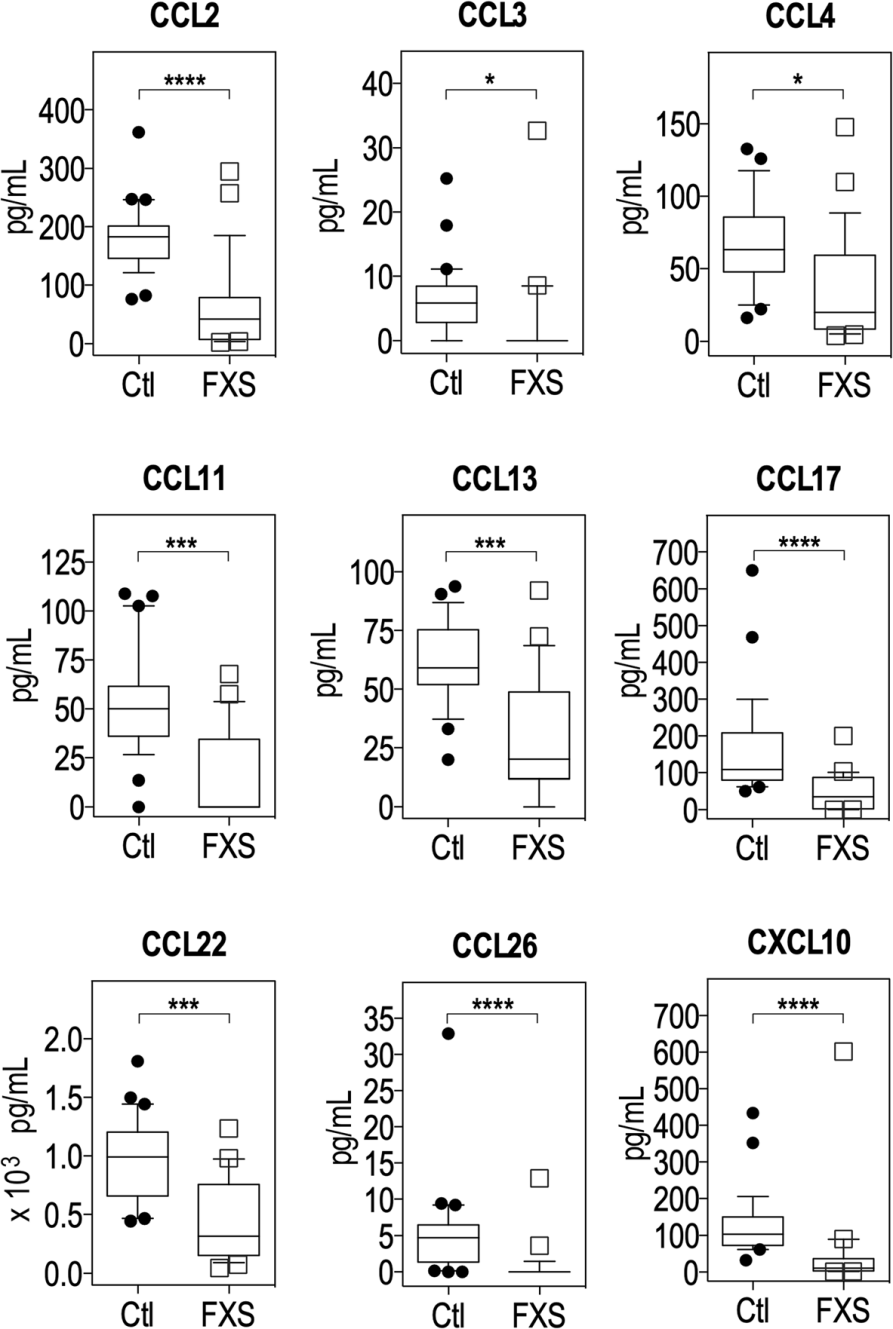
Levels of chemokines downregulated in FXS patients as compared to controls. Data are presented as a box-and-whiskers plot; *n* = 29 controls, *n* = 25 FXS patients. The box plots the first quartile, median and third quartile of the values. The whiskers are drawn down to the 10th percentile and up to the 90th. Points below and above the whiskers are drawn as individual dots. Statistical significance of differences was measured using the Mann–Whitney U-test based on adjusted *p-*values. *: *p* < 0.01; ***: *p* < 0.001; ****: *p* < 0.0001

We have analysed serum samples of 25 FXS patients (*n* = 20 males; *n* = 5 females) and 29 healthy controls (*n* = 24 males; *n* = 5 females) (Table 1). The two groups did not differ in terms of sex proportions, age, BMI or time of sampling (Table 1). Fifty-two cytokines and inflammation and tissue damage markers were quantified. Out of these biomarkers, 6 cytokines (IL-1α, IL-1β, IL-2, IL-4, IL-5 and IL-13) were below the lower limit of detection in more that 20% of control samples (Additional file 2: Table S1) and were therefore not retained for downstream analysis. We then used univariate statistical methods to compare the levels of the remaining biomarkers in FXS patients and healthy controls. The majority of these biomarkers were present at similar levels in patients and controls (Additional file 3: Table S2). In contrast, 10 biomarkers were differentially expressed between FXS patients and controls (*p*-value < 0.05, Additional file 3: Table S2): CCL2, CCL3, CCL4, CCL11, CCL13, CCL17, CCL22, CCL26, CXCL10 and IL-15. After multiple testing correction, only the 9 chemokines appeared significantly decreased in FXS patients, as compared to controls (adjusted *p*-value < 0.05, Additional file 3: Table S2, Fig. 1). Large effect sizes (0.5 < *r* coefficient < 0.8) were observed for all dysregulated chemokines except for CCL4 in which the effect size was medium (0.2 < *r* coefficient < 0.5) (Additional file 3: Table S2). The same 10 biomarkers were significantly dysregulated in the dataset reduced to male subjects (*p*-value < 0.05, Additional file 3: Table S2). After multiple testing correction, seven out of the 9 chemokines identified in the full dataset (CCL2, CCL11, CCL13, CCL17, CCL22, CCL26 and CXCL10) were also significantly reduced in FXS male patients as compared to male controls (adjusted *p*-value < 0.05, Additional file 3: Table S2). Although the small number of females in our dataset (*N* = 10 out of *N* = 54 subjects) precluded a clear analysis of the effect of sex, the later results suggest a minimal impact of sex on chemokine levels.

To ascertain the results of univariate statistics, we conducted a multivariate analysis using robust Elastic Net penalized regression to evaluate the association between chemokine levels and FXS diagnosis, adjusting for covariates possibly impacting the levels of chemokines (age, BMI, time of sampling). As compared to non-robust Elastic Net regression, robust regression presented the advantage of downweighing the effect of extreme values and potential outliers in terms of biomarkers’ concentrations, which could affect the stability of the analysis (Fig. 1, Additional file 3: Table S2). This analysis revealed that the levels of CCL2, CCL3, CCL11, CCL22, CCL26 and CXCL10 were negatively associated with increased odds of FXS diagnosis, while the covariates age, BMI and time of sampling were not (Table 2). Sex being another possible confounding factor and as robust regression currently does not handle categorical variables, we rerun the robust regression analysis on a sample restricted to male individuals. We confirmed that 5 chemokines CCL2, CCL11, CCL22, CCL26 and CXCL10 remained stably associated with increased odds of FXS diagnosis in the dataset restricted to male subjects, reflecting a minimal impact of sex on these associations (Table 2). However, CCL4, CCL13 and CCL17 appeared associated with FXS diagnosis only in the dataset restricted to males. This supports the stability of our results for CCL2, CCL11, CCL22, CCL26 and CXCL10 and possible sex differences in the expression of chemokines for CCL3, CCL4, CCL13 and CCL17.

**Table 2:** Associations between chemokines and FXS diagnosis adjusted for age, BMI and time of sampling

	Dataset	
	Males + Females (*N* = 54)	Males (*N* = 44)
**Covariates**		
Age	0	0
Time of sampling	0	−0.04093
BMI	0	−0.15352
**Chemokines**		
**CCL2**	**−0.03703**	**−0.05208**
CCL3	−0.01263	0
CCL4	0	0.06623
**CCL11**	**−0.01220**	**−0.01161**
CCL13	0	−0.05315
CCL17	0	−0.00101
**CCL22**	**−0.00060**	**−0.00743**
**CCL26**	**−0.04703**	**−1.04540**
**CXCL10**	**−0.01051**	**−0.03173**

Coefficients were obtained using robust Elastic Net regression with 2000 subsampling steps on the whole dataset (males and females subjects: *N* = 54, *n* = 25 FXS patients, *n* = 29 controls) and in a dataset reduced to male subjects (*N* = 44, *n* = 20 FXS patients, *n* = 24 controls). Coefficients distinct from zero are shaded in grey and convergent associations across the two datasets are bolded

While some chemokines bind to several receptors, some chemokine receptors bind different chemokines [16]. To gain further insight into the immune pathways that could be impacted by chemokine dysregulation, we constructed an interaction map using available databases (see Material & Methods for extensive list of sources). We identified six chemokine receptors, CCR1, CCR2, CCR3, CCR4, CCR5 and CXCR5, whose signalling could be impacted (Additional file 1: Fig. S1A). We then performed a pathway over-representation analysis on the list of the nine downregulated chemokines in FXS patients (Additional file 1: Fig. S1B*,* Additional file 4: Table S3). Biological processes related to chemotaxis of innate and adaptive immune cell types were over-represented: CCL2 and CCL3 for lymphocytes, monocytes, macrophages and neutrophils, CCL3, CCL11 and CCL13 for eosinophils, neutrophils, granulocytes, CCL3 and CXCL10 for T cells, CCL3 and CCL4 for NK cells. Several chemokines were also involved in response to viral infection (CCL4, CCL11, CXCL10), bacterial infection (CCL2, CCL3, CXCL10), as well as to toxic substances insults (CCL3, CCL4).

### Clustering analysis

**Fig. 2:**
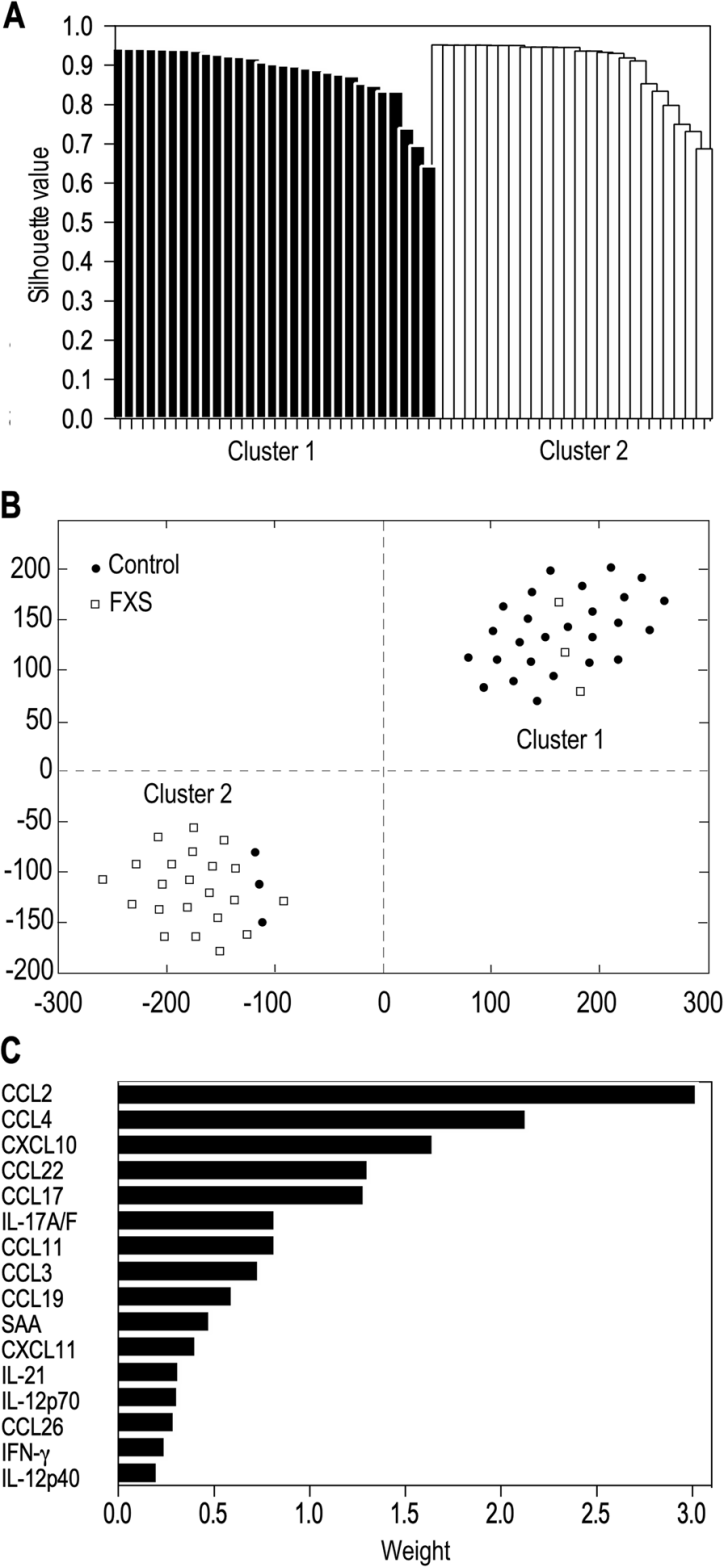
K-sparse clustering analysis reveals clear discrimination of FXS patients from healthy controls based on biomarkers levels. **a** t-Distributed Stochastic Neighbour Embedding (*t-SNE*) graphical output of K-sparse clustering analysis with 2 clusters. **b** Performance of the model evaluated using the individual silhouette scores. Each column represents one individual and its individual silhouette value evaluating its parenthood with underlying cluster. **c** Combination of discriminating features for K-sparse clustering, with their corresponding norm of weights (norm of their associated row in the *W* matrix, see Additional file 6: Table S5)

In contrast to univariate methods that assess the differential expression of cytokines at the single feature level, unsupervised clustering methods allows for identifying combinations of variables with the best joint discriminative ability to separate the classes of samples. We therefore applied unsupervised classification to the dataset encapsulating the levels of the studied biomarkers for each of the 54 individuals included in our study. To partition the samples in two clusters, we used the K-sparse algorithm which not only identifies underlying homogeneous clusters but also selects the combination of biomarkers relevant to discriminate each cluster. We have previously proven the superior efficacy of K-sparse clustering towards more classical PCA k-means clustering [24]. In order to assess the quality of this K-sparse partitioning in two clusters, we computed the silhouette value for each patient. Silhouette value measures how similar a patient is to the patients in its own cluster (i.e. the intra-cluster cohesion), compared to patients in other clusters (i.e. the inter-cluster separation). The closest the silhouette coefficient (average of all the silhouette values) approaches 1, the better the intra-cluster consistency is. The mean silhouette coefficient associated to our clustering was equal to 0.892 and most of the individual silhouette values were close to 1 (Fig. 2a, Additional file 5: Table S4), which indicated the appropriateness of our clustering and which revealed that the 2 clusters are well distinct in the projected space (Fig. 2b). Since clustering analysis was unsupervised, the model was not a priori informed of the samples class (i.e. Control or FXS). We therefore compared our clusters labels to these class’ labels (Fig. 2b). Among the 29 individuals in Cluster 1, 26 were healthy controls and 3 were FXS patients. In contrast, among the 25 individuals in Cluster 2, 22 were FXS patients and 3 were healthy controls. This revealed that our partitioning matches well with the two classes Control and FXS.

The procedure of features selection proposed by K-sparse algorithm allowed for identifying relevant biomarkers that could discriminate the two clusters using the weighted projection matrix *W* associated to this clustering (Additional file 6: Table S5). K-sparse clustering discriminated healthy controls from FXS patients on the basis of the combination of 16 cytokines (Fig. 2c). This combination of biomarkers overlaps with the list of chemokines displaying the largest significant changes (CCL2, CCL3, CCL4, CCL11, CCL17, CCL22, CCL26, CXCL10, Fig. 1) to the exception of CCL13 which was not selected (Fig. 1, 2c). It also highlighted that CCL19, CXCL11, IL-12p40, IL-12p70, IL-17A/F, IL-21, INF-γ and SAA have discriminative capacities, although these markers were not differentially expressed between FXS and controls in univariate analysis (Fig. 2c).

**Fig. 3:**
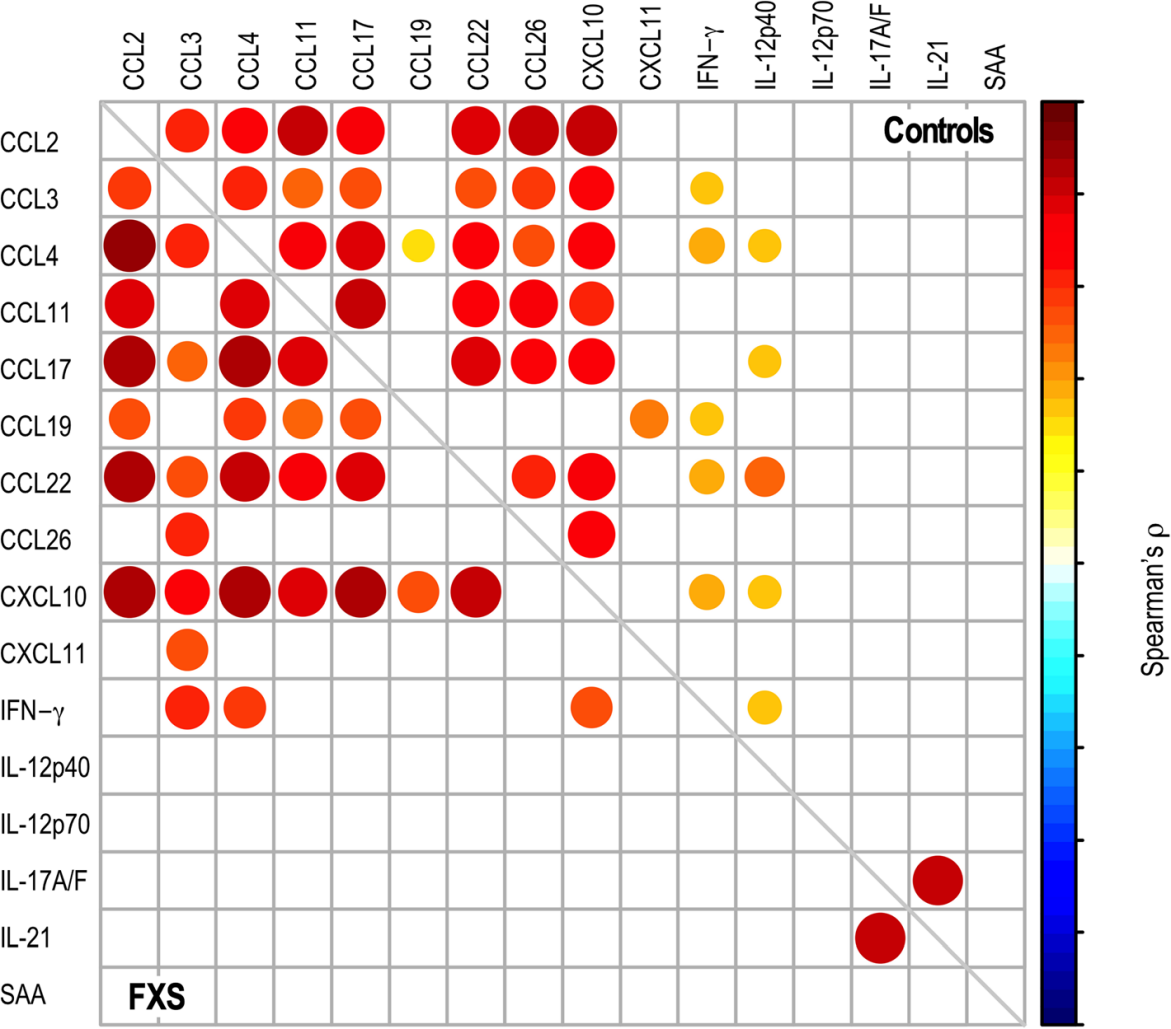
Correlation analysis of the serum levels of the 16 discriminating cytokines identified by K-sparse clustering in healthy controls and FXS patients. Mirror heatmap representation of correlations. Spearman’s ρ coefficients are color-coded and proportional to dot area. Only significant correlations are displayed (adjusted *p*-value < 0.05)

To identify potential relationships between these 16 biomarkers, we performed correlation analyses in healthy controls and FXS patients respectively (Fig. 3, Additional file 7: Table S6). IL-12p70, IL-17A/F, IL-21 and SAA did not display significant correlations with any of the markers in both controls and patients, to the exception of the IL-17A/F and IL-21 interleukin pair (Fig. 3). Similar correlation patterns were observed in control individuals and in FXS patients for combinations of the chemokines CCL2, CCL3, CCL4, CCL11, CCL17, CCL22 and CXCL10. In contrast, CCL19, CCL26, IL-12p40 and IFN-γ displayed divergent patterns of correlation. Medium to strong positive correlations were observed in FXS patients for the combinations of 7 chemokines (CCL2, CCL3, CCL4, CCL17, CCL19, CCL22 and CXCL10) and IFN-γ correlated with CCL3, CCL4 and CXCL10. This suggests the presence of coordinated variations in the levels of those biomarkers in FXS patients (Fig. 3).

## Discussion

### Reduced levels of pro-inflammatory chemokines in FXS patients

Compared to healthy individuals, Ashwood et al. found that FXS patients with ASD exhibited higher plasma levels of IL-1α and IL-12p40 and reduced levels of CCL2, CCL5, CCL11 and CXCL10, but similar levels of IL-1β, IL-2, IL-3, IL-4, IL-5, IL-6, IL-7, IL-8, IL-10, IL-12p70, IL-13, IL-15, GM-CSF, IFN-γ, TNF-α and CCL3 [17]. They also found that FXS patients without ASD exhibited higher plasma levels of IL-1α and reduced levels of CCL5 and CXCL10, but similar levels of the other biomarkers [17]. While seven interleukins, i.e. IL-1α, IL-1β, IL-2, IL-4, IL-5 and IL-13 were readily detected in plasma samples by Ashwood et al. using a different immunoassay, these IL were below the lower level of detection in our serum samples. In agreement with this previous study, we found that IL-6, IL-7, IL-8, IL-10, IL-12p40, IL-12p70, IL-15, GM-CSF, IFN-γ and TNF-α were present at similar levels in FXS patients and healthy controls. However, we did not highlight an increase in IL-12p40 in FXS patients and we show a decrease in CCL3. These apparent discrepancies could be explained by the type of matrix used (plasma versus serum), the analytical platform used (bead-array versus ECL assay) or differences in the statistical methods used. However, both our study and the one by Ashwood et al. suggest that FXS patients do not exhibit a clear low-grade pro-inflammatory profile that would be characterized by higher levels of multiple pro-inflammatory cytokine and acute phase proteins such as CRP. In agreement with this observation in FXS patients, serum levels of the two pro-inflammatory cytokines TNF-α and IFN-γ were reported to be identical in *Fmr1*-KO mice and their non-transgenic littermates [25].

Chemokines are well known for their ability to induce directed chemotaxis in nearby responsive cells. Here, we have found that FXS patients exhibit reduced serum levels of CCL2, CCL3, CCL4, CCL11, CCL13, CCL17, CCL22, CCL26 and CXCL10. Furthermore, CCL2, CCL3, CCL11, CCL22, CCL26 and CXCL10 were negatively associated with FXS diagnosis in a robust regression model in which we adjusted for age, BMI and time of sampling. Five of these chemokines (CCL2, CCL11, CCL22, CCL26 and CXCL10) were also retained in a dataset reduced to male subjects, strengthening our findings for these chemokines. Finally, CCL2, CCL3, CCL11, CCL22, CCL26 and CXCL10 are also selected by our K-sparse clustering as features discriminating the clusters, in combination with 10 additional biomarkers. Importantly, a previous report highlighted that the plasmatic levels of CCL2, CCL11 and CXCL10 were significantly decreased in a larger sample of male FXS patients [17]. All these data support the fact that dysregulation of at least a subset of specific pro-inflammatory chemokines could contribute to FXS.

The subset of chemokines exhibiting reduced levels in FXS patients indicated possible decreased signalling from six chemokine receptors: CCR1, CCR2, CCR3, CCR4, CCR5 and CXCR3. This could possibly impact the chemotaxis of a number of cell types and pathways ontologically related to “inflammatory response”, including “response to viral infection”, “response to LPS” or “response to toxic insults”. CXCR3 is expressed primarily by activated NK cells and T lymphocytes, and by epithelial cells [26]. Th1 cells co-express CXCR3 and CCR5 while Th2 cells express CCR3 and CCR4. CXCR3 ligands that attract Th1 cells can concomitantly block the migration of Th2 cells in response to CCR3 ligands, thus enhancing the polarization of effector T cell recruitment [26]. Reduced serum levels of CXCL10 might dampen CXCR3 signalling, while decreased levels of CCL3 and CCL5 could reduce CCR3 and CCR4 signalling. This could ultimately cause T cell dysfunction in FXS patients. CCL2 is the most strongly dysregulated chemokine in FXS patients as compared to controls. Through signalling via CCR2 and CCR4 receptors, CCL2 mediates chemotaxis of monocytes and dendritic cells, as well as memory T cells to the sites of inflammation upon tissue injury or infection [27, 28]. Decreased levels of circulating CCL2 could therefore contribute to reduce the local immune response to infection in FXS patients.

A general health survey on FXS patients highlighted an increased occurrence of ear, throat and nose infections such as sinusitis and otitis in FXS patients [9, 10]. However, anatomical particularities and malformations of the ear conducts and sinuses are likely to explain the specificity of these infections [9], as FXS patients do not appear more sensitive to other types of infections. This would have been expected, if there was a general defective chemokine receptor signalling during infection in FXS patients. Nevertheless, it may be envisioned that reduced chemokine levels in FXS patients may be associated with a reduced capacity of the immune system of FXS patients to respond to specific pathogens. Of note, one study in the *Drosophila melanogaster* model of FXS showed that the *dfmr1* mutants exhibit increased sensitivity to bacterial infection and decreased phagocytosis of bacteria by systemic immune cells [29], suggesting that *dfmr1* gene is required for the activation of phagocytic immune cells and therefore for their immune responsiveness.

Although immune responsiveness to LPS and PHA of PBMC from FXS patients did not differ from those of healthy controls [18], a few studies have been performed in asymptomatic individuals carrying the *FMR1* premutation. Notably, an increase in IL-10 secretion by PBMC in the absence of immune challenge was described in *FMR1* premutation carriers [30]. Furthermore, a decreased cytokine secretion response was observed in LPS-stimulated PBMC from individuals carrying the *FMR1* premutation [31]. This suggests that dysregulation in the *FMR1* gene could alter immune responsiveness under yet to be defined conditions. FMRP, the *FMR1* gene product, is an RNA-binding protein and a translational regulator [6]. Although majorly described as a translational repressor, some studies suggest that FMRP can activate the translation of some of its mRNA targets [6]. Further work would be required to determine whether the mRNAs encoding the six dysregulated cytokines are bona fide mRNA targets for translational activation by FMRP or whether their decreased levels indirectly result from compensatory or adaptive mechanisms.

### Possible neuro-immune alterations in FXS

Interactions between the nervous and the immune system are critical not only during early neurodevelopment but also in adolescence and adulthood [32]. As a consequence, immune dysfunction may cause changes in brain connectivity associated with neurodevelopmental disorders and this has been mostly studied in the context of ASD [33]. Chemokine and their cognate receptors are widely expressed in the developing and adult CNS and disruption of their patterns of expression have been involved in CNS disorders, including ASD [34]. Notably, CCL2, CCL3, CCL4, CCL5 and CCL11 are required, via signalling through CCR2, CCR3 and CCR5 receptors, for microglia chemotaxis [35–37]. In addition, CCL2 regulates migration of neural stem cells in the brain [38]. Although peripheral levels of those chemokines might not reflect their actual levels in the CNS, alterations in CCL2, CCL3, CCL4, CCL5 and CCL11 signalling could participate to the defects in CNS patterning observed in FXS patients. In addition, alterations in chemokine secretion or immune response could enhance the sensitivity of FXS patients to neurological damages induced by environmental sources, including infections and xenobiotics exposure. In line with this, and although chemokines were not assessed, one study has shown that cortical astrocytes derived from the brain of FXS mouse model secreted more IL-6 in response to LPS stimulation. Furthermore, the authors provide evidence that the abnormal elevation of IL-6 in the cortex of *Fmr1*-KO mouse could be linked to the synaptic phenotypes [39].

### Immune-related biomarkers enable clustering of samples in FXS and control cases

The K-sparse clustering strategy we applied to the biomarkers’ dataset enabled discrimination of FXS samples from control samples relying principally on a combination of 16 immune-related markers. A number of efficient methods already exist to perform unsupervised classification of samples based on datasets encapsulating biological features. It is common practice to use PCA k-means to perform clustering analysis. k-means does not perform both clustering and feature selection, therefore providing minimal insights into the discriminating features and therefore into the underlying biology. The K-sparse method we have used alternates k-means with projection-gradient minimization to promote sparsity and enable features selection, with significant improvements in clustering performances as compared to k-means standard algorithm in terms of clustering performances [24]. By considering the weighted linear combination of the selected features, the clustering computed by K-sparse also presented the advantage of handling extreme values and outliers which are frequently observed in biomarker datasets. The K-sparse clustering highlighted that 8 of the 9 significantly dysregulated chemokines contribute to separation between controls and FXS cases. It further identified additional immune-related molecules with discriminative abilities: CCL19, CXCL11, IL-12p40, IL-12p70, IL-17F, IL-21, INF-γ and SAA. K-sparse highlights the contribution of biomarkers which do not differ in univariate analysis and regression analysis. This can be explained by the fact that K-sparse relies on weighted linear combination of the selected features and not on the individual distribution of values among each class. Correlations are observed between a subset of the 16 biomarkers selected by K-sparse, showing that relationships between biomarkers can be of biological relevance. Our study supports that K-sparse clustering can complement classical univariate and regression analyses to identify relevant biomarkers of disease. It paves the way for the use of K-sparse clustering analysis for the identification of combinations of disease biomarkers, but also for the stratification of patients in homogenous subtypes bearing similar biological patterns.

### Limitations of the study

Our study has some limitations. First, the sample size is relatively small (*n* = 25 FXS patients; *n* = 29 sex and age-matched controls), which may lead to an overestimation of the effect size owing to reduced power, but also limit the generalization of our findings. Second, the methodological choices we have made based on out dataset constraints, e.g. the use of robust Elastic Net regression instead of classical logistic regression, may limit the interpretation of the identified associations. Indeed, coefficients are shrunk and computation of confidence interval and asymptotically valid *p*-values are not yet available in the robust regression framework. However, among the six chemokines that we have shown here to be negatively associated with FXS, three (CCL2, CCL11, CXCL10) have already been demonstrated to be present at lower levels in an independent cohort of FXS patients (*n* = 64) compared to healthy controls (*n* = 19) [17], therefore strengthening the validity of our conclusions. Third, although we have adjusted the associations between specific chemokines and FXS diagnosis for a number of covariates which could impact serum cytokines (age, BMI and time of sampling), we cannot rule out the contribution of additional unmeasured covariates. Fourth, we acknowledge that the present study provides limited insight into possible underlying mechanisms. However, it is noteworthy that almost nothing is known on possible immune-related dysfunctions in FXS patients. Exploratory studies are therefore needed to provide the rationale for new studies investigating specific underlying mechanisms.

## Conclusions

Our data suggest that immune dysfunction could participate to the physio-pathological processes involved in FXS. The dysregulated immune markers are pro-inflammatory chemokines which are reduced, suggesting that inflammation is not a hallmark of FXS. Further studies are required to decipher the possible role played by immune molecules, and in particular chemokines, in the pathophysiology of FXS and other neurodevelopmental disorders.

## Supplementary information

**Additional file 1: Figure S1.** Pathways associated with significantly dysregulated chemokines in FXS patients. (A) Interaction map for the subset of chemokines dysregulated in FXS patients and their cognate receptors CCR1, CCR2, CCR4, CCR5 and CXCR10. (B) Selection of GO terms significantly over-represented in the list of dysregulated chemokines presented in Fig. 1.

**Additional file 2: Table S1.** Percentage of detection of biomarkers in FXS patients vs. healthy controls and inclusion of analytes

**Additional file 3: Table S2.** Comparison analysis of serum biomarkers levels in FXS patients and controls in the whole dataset (*N* = 54) and in a reduced dataset consisting of male individuals (*N* = 44). Minimum (Min), first quantile (Q1), median, third quantile (Q3), maximum (Max), mean, standard deviation (SD) are indicated for each group. The *r* effect size statistic, raw *p*-values (*p*-value) and adjusted *p-*values for the Mann–Whitney U-test, are indicated both for the full dataset (N = 54, *n* = 25 FXS patients, *n* = 29 controls) and for the dataset reduced to male individuals (N = 44, *n* = 20 FXS patients, *n* = 24 controls).

**Additional file 4: Table S3.** Over-represented pathways based on the list of significantly downregulated chemokines in FXS patients

**Additional file 5: Table S4.** Silhouette values for each individual in the K-sparse clustering analysis with k = 2 and clustering accuracy based on samples class

**Additional file 6: Table S5.***W* matrix highlighting the respective discriminative weight of each feature in the 4 dimensions of the K-sparse model

**Additional file 7: Table S6.** Correlations and associated adjusted p-values for each biomarker pair among the 16 features identified by K-sparse clustering in healthy controls and FXS patients. For each biomarker pair, correlations *r* coefficients and the corresponding adjusted p-values are presented in upper triangle and lower triangle, respectively. Adjusted *p*-values < 0.05 are highlighted in bold.

## Data Availability

The datasets used and analysed during the current study as well as the complete code for K-sparse clustering are available from the corresponding author on reasonable request.

## Abbreviations

FXS: Fragile X Syndrome
CNS: central nervous system
*FMR1*: Fragile X Mental Retardation 1 gene
IL: interleukin
LPS: lipopolysaccharides
PBMC: peripheral blood mononuclear cells
ASD: autism spectrum disorder
TNF: Tumor Necrosis Factor
IFN: interferon
