# Supplementary figures and images for: Reduced serum levels of pro-inflammatory chemokines in fragile X syndrome
Source: BMC Neurol. 2020 Apr 15;20:138. doi: 10.1186/s12883-020-01715-2 (PMC7161166; doi:10.1186/s12883-020-01715-2)

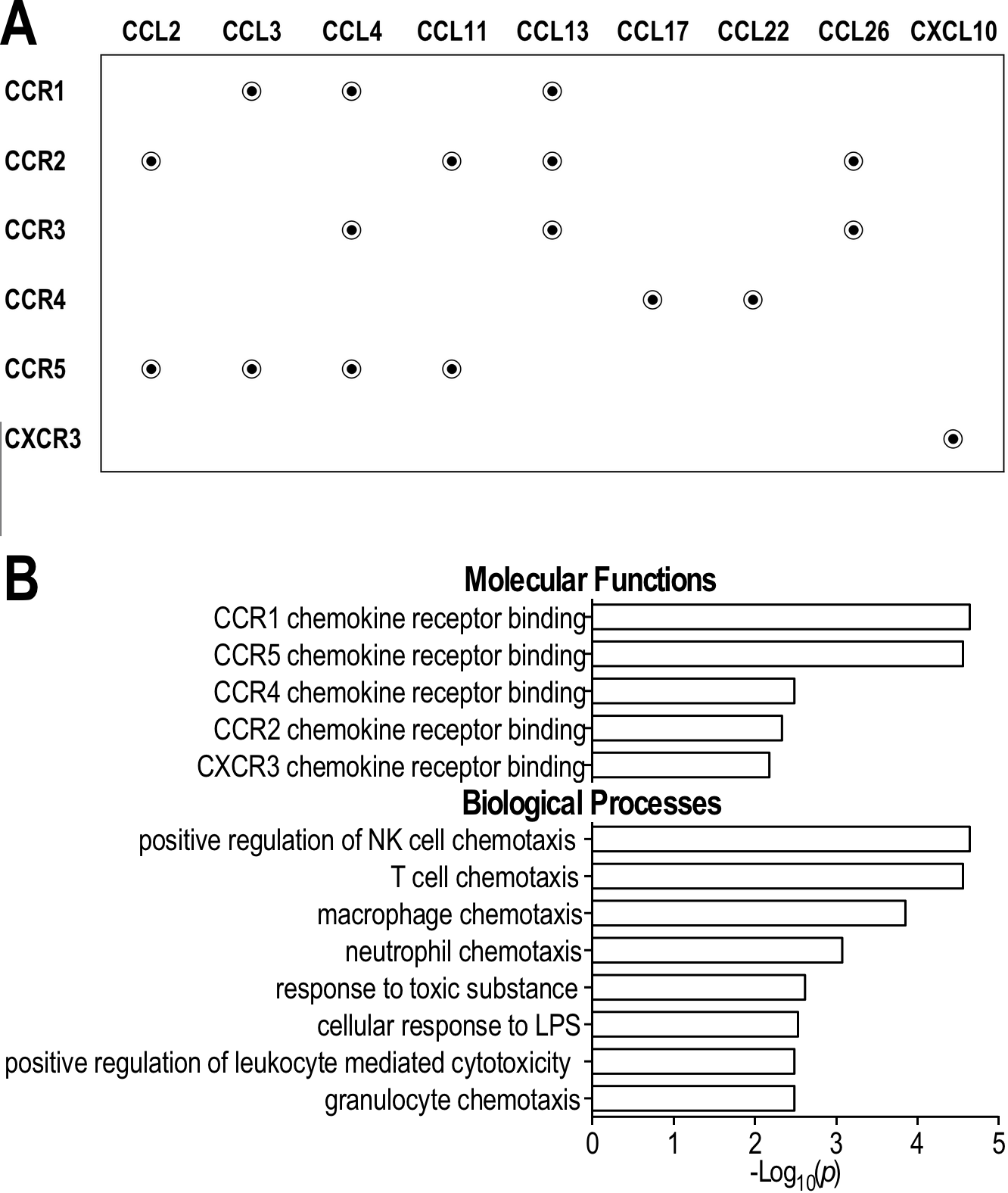

Supplement: Supplementary file 1 — Additional file 1: Figure S1. Pathways associated with significantly dysregulated chemokines in FXS patients. (A) Interaction map for the subset of chemokines dysregulated in FXS patients and their cognate receptors CCR1, CCR2, CCR4, CCR5 and CXCR10. (B) Selection of GO terms significantly over-represented in the list of dysregulated chemokines presented in Fig. 1. [file 12883_2020_1715_MOESM1_ESM.tif]
